# Investigation of DNA repair-related SNPs underlying susceptibility to papillary thyroid carcinoma reveals *MGMT* as a novel candidate gene in Belarusian children exposed to radiation

**DOI:** 10.1186/s12885-017-3314-5

**Published:** 2017-05-12

**Authors:** Christine Lonjou, Francesca Damiola, Monika Moissonnier, Geoffroy Durand, Irina Malakhova, Vladimir Masyakin, Florence Le Calvez-Kelm, Elisabeth Cardis, Graham Byrnes, Ausrele Kesminiene, Fabienne Lesueur

**Affiliations:** 1 Institut Curie, 75248 Paris, France; 2 PSL Research University, 75005 Paris, France; 3 INSERM, U900, 75248 Paris, France; 4 Mines Paris Tech, 77305 Fontainebleau, France; 5 Biopathologie, Centre Léon Bérard, 69373 Lyon, France; 6 Environment and Radiation, International Agency for Research on Cancer (IARC), 69372 Lyon, France; 7 Genetic Cancer Susceptibility, IARC, 69372 Lyon, France; 8 Republican Scientific and Practical Center for Medical Technologies, Informatisation, Administration and Management of Health (RSPC MT), 220013 Minsk, Belarus; 9 Republican Research Center for Radiation Medicine & Human Ecology, 246040 Gomel, Belarus; 10 Centre for Research in Environmental Epidemiology (CREAL), IMIM (Hospital del Mar Research Institute), CIBER Epidemiología y Salud Pública (CIBERESP), 08003 Barcelona, Spain

**Keywords:** Papillary thyroid carcinoma, Radiation-induced cancer, Genetic susceptibility, DNA repair, *MGMT*

## Abstract

**Background:**

Genetic factors may influence an individual’s sensitivity to ionising radiation and therefore modify his/her risk of developing papillary thyroid carcinoma (PTC). Previously, we reported that common single nucleotide polymorphisms (SNPs) within the DNA damage recognition gene *ATM* contribute to PTC risk in Belarusian children exposed to fallout from the Chernobyl power plant accident. Here we explored in the same population the contribution of a panel of DNA repair-related SNPs in genes acting downstream of ATM.

**Methods:**

The association of 141 SNPs located in 43 DNA repair genes was examined in 75 PTC cases and 254 controls from the Gomel region in Belarus. All subjects were younger than 15 years at the time of the Chernobyl accident. Conditional logistic regressions accounting for radiation dose were performed with PLINK using the additive allelic inheritance model, and a linkage disequilibrium (LD)-based Bonferroni correction was used for correction for multiple testing.

**Results:**

The intronic SNP rs2296675 in *MGMT* was associated with an increased PTC risk [per minor allele odds ratio (OR) 2.54 95% CI 1.50, 4.30, *P*_per allele_ = 0.0006, *P*_*corr.=*_0.05], and gene-wide association testing highlighted a possible role for *ERCC5* (*P*_Gene_ = 0.01) and *PCNA* (*P*_Gene_ = 0.05) in addition to *MGMT* (*P*_Gene_ = 0.008).

**Conclusions:**

These findings indicate that several genes acting in distinct DNA repair mechanisms contribute to PTC risk. Further investigation is needed to decipher the functional properties of the methyltransferase encoded by *MGMT* and to understand how alteration of such functions may lead to the development of the most common type of thyroid cancer.

**Electronic supplementary material:**

The online version of this article (doi:10.1186/s12885-017-3314-5) contains supplementary material, which is available to authorized users.

## Background

Thyroid cancer (TC), with an age-standardised incident rate of 4.0 per 100,000 men and women in developed countries (http://ci5.iarc.fr) is the most common type of endocrine malignancy and it is now the fifth most common cancer diagnosed in women [1]. Papillary thyroid carcinoma (PTC) along with follicular thyroid carcinoma (FTC) and Hürthle cell carcinoma (a subtype of FTC) is termed differentiated thyroid carcinoma (DTC). All together these TC types account for more than 90% of all TC and PTC is the most common subtype, representing approximately 80% of cases. During the last three decades incidence rates of TC, and in particular of PTC, have increased in nearly all countries [2]. This trend is partly attributable in high-resource countries to increased surveillance of the thyroid gland and improved detection methods [3], but it is also possible that changes in exposure to environmental factors and ionizing radiation, a well-established risk factor for this cancer especially when it occurs during childhood or as a young adult, could influence PTC incidence [4–6]. Other risk factors for PTC include iodine deficiency and excess [7], previous history of benign thyroid disease, such as nodules and autoimmune thyroid disease, as well as a family history of DTC. Remarkably, DTC has a strong familial component and first-degree relatives of DTC patients have up to eight times higher risk of developing DTC than the general population, indicating that genetic factors play an important role in DTC risk [8, 9]. The observed familial risk could be partly explained by high-penetrance mutations in yet unidentified genes or by the additive effect of numerous low-penetrance variants [10, 11]. This latter hypothesis would explain the paucity of families with more than two affected members. Recent case-control studies, in particular Genome-Wide Association Study (GWAS) have highlighted a number of low-penetrance alleles contributing to sporadic DTC risk. The association of DTC with the 9q22 (rs965513) locus close to the thyroid specific factor *FOXE1* has been detected in all association studies conducted so far in different populations [12]. Weaker associations have been reported, among others, with rs944289 at 14q13.3 (near *NKX2–1*), rs966423 at 2q35 (in *DIRC3*), rs334725 at 1p31.3 (in *NFIA*) and rs2439302 at 8p12 (in *NRG1*) [12–14]. Since the associations described so far only explain about 10% of the familial risk of DTC [15], additional studies on specific populations are of particular relevance, especially studies of high-risk populations having been exposed to known environmental risk factors. Notably a sharp increase in incidence of thyroid malignancies, virtually all PTC, has been observed in children and adolescents that were exposed to radioactive fallout from the Chernobyl nuclear power plant accident in April 1986. Variation in clinical course, ranging from highly aggressive tumours developing after the shorter latency to more indolent carcinomas with longer latent period has been reported in this population [16], which suggests that some predisposing genetic factors could influence an individual’s sensitivity to radiation and therefore modify the risk of developing TC in exposed population [17, 18].

DNA repair is an important defence mechanism against DNA damage caused by normal metabolic activities and environmental factors [19]. DNA damage is recognized and processed by a series of distinct pathways called the “DNA damage response (DDR)” [20]. It includes direct repair (DR), base and nucleotide excision repair (BER and NER), mismatch repair (MMR), double strand break repair (DSBR) and interstrand cross-links repair system [21, 22]. Because ionizing radiation produces DNA lesions and DNA repair genes play a critical role in maintaining genome integrity, it has been proposed that inherited variations in such genes may reduce DNA repair capacity and influence cancer development in exposed subjects. Few case-control studies on radiation-related PTC have been conducted to date and published data suggest that alterations in expression levels and polymorphisms in some candidate DNA repair genes belonging to the different pathways are implicated in risk of developing thyroid cancer [23–26]. In particular, others and we have reported association with the coding SNP rs1801516 (p.Asp1853Asn) in the *ATM* gene, a key regulator of signalling following DNA double-strand breaks (DSBs) [25–27]. Other possibly involved DNA repair-related SNPs include rs25487 (p.Arg399Gln) in *XRCC1* involved in BER [25, 27–29] and rs13181 (p.Lys751Gln) in *ERCC2* involved in NER [30]. To follow up with these findings, we hypothesized that alterations in other genes related to the different DNA repair mechanisms may be correlated with tumour susceptibility in subjects originated from the Gomel region of Belarus and having been exposed to radioactive fallout from the Chernobyl nuclear power plant accident during childhood. Here we evaluated the risk association between SNPs in DNA repair genes present on the Cancer SNP panel array (Illumina) and PTC in this very unique population. Findings of this study may increase understanding of PTC aetiology and help identify subjects who may be particularly susceptible to the carcinogenic effects of radiation on the thyroid.

## Methods

### Study population

A total of 83 PTC cases and 324 matched and unrelated controls living in the Gomel region, one of the most contaminated areas of Belarus, were included in the present study. Participants correspond to a sub-group of subjects from the population-based case-control study carried out at the International Agency for Research on Cancer to evaluate the risk of thyroid cancer after exposure to radioactive iodine in childhood [17]. As we reported previously [26], all subjects “were younger that 15 years at the time of the Chernobyl accident” and “the control subjects were matched to the PTC cases by age (within 1 year for those who were 18 months or older at the time of the accident; within 6 months for those who aged 12-18 months; and within 1 month for who were younger than 12 months) and sex. The cases were diagnosed within 6 to 12 years following the accident with histologically verified PTC, mostly solid/follicular subtype, confirmed by the international panel of pathologists. Two thirds of the cases developed PTC before the age of 15 and the remaining cases before the age of 25. For more than 60% of cases, the latency (time between radiation exposure and diagnosis) was less than 10 years” [26] (Table 1). Individual radiation dose to the thyroid was reconstructed based on the study participant’s whereabouts and dietary habits, and information on environmental contamination for each settlement [17, 26, 31]. The radiation dose-response was similar for subjects included in the current analysis (β = 1.51, 95% CI 0.46–2.55) compared to those who did not consent to blood drawing (β = 1.55, 95% CI 1.04–2.07) [26].

**Table 1:** Characteristics and distribution of study participants

	Cases	Controls	Total
Gender			
Male	35 (42%)	127 (39%)	162
Female	48 (58%)	197 (61%)	245
Age at exposure			
< 1 y	9 (11%)	73 (22.5%)	82
1–1.9 y	14 (17%)	46 (14%)	60
2–2.9 y	9 (11%)	40 (12%)	49
3–3.9 y	17 (20%)	40 (12%)	57
4–4.9 y	9 (11%)	43 (13%)	52
5–5.9 y	4 (5%)	18 (5.5%)	22
6–9 y	13 (15%)	35 (11%)	48
10–14 y	8 (10%)	29 (10%)	37
Age at diagnosis			
< 11.5 y	24 (29%)	119 (37%)	143
11.5–14.9 y	33 (40%)	120 (37%)	153
15+ y	26 (31%)	85 (26%)	111

The study population was rural and highly dependent on the local food produced in known iodine deficient areas [32]. As previously described in this population, “the level of stable iodine intake was correlated with the iodine concentration in the agricultural lands around their places of residence” [33], and “stable iodine intake status was estimated for each individual as a crude index based on the average level of stable iodine soil content in the settlement of residence of the study subject at the time of the Chernobyl accident” [33].

### Genotyping

Genomic DNA was extracted from peripheral blood samples using a standard inorganic method [34] as previously described [26].

Study participants were genotyped for a total of 1421 SNPs located in 407 genes involved in cancer-related pathways using the Illumina GoldenGate Assay (Illumina Inc., USA) according to the manufacturer’s recommendations. The Cancer SNP Panel array contains SNPs within genes involved in the aetiology of various types of cancer selected from the National Cancer Institute’s Cancer Genome Anatomy Project SNP500Cancer Database [35]. It contains more than 3 SNPs, on average, for each gene represented on the panel. The complete list of the annotated SNPs present on the array is provided in Additional file 1: Table S1.

### Selection of SNPs in DNA repair related genes

For the purpose of this study, we chose to focus the analysis of the genotyping data on candidate SNPs located within genes involved in DNA repair pathways as annotated in the Atlas of Cancer Signalling Network (ACSN) [36]. According to the ACSN, 178 out of the 1421 SNPs present on the Cancer SNP Panel array are located in a DNA repair gene. Among the 178 SNPs, 141 passed the genotyping quality controls (QCs) and had a minor allele frequency (MAF) in the control group greater than 0.05; those SNPs were included in the analyses. The list of the 141 analysed SNPs is accessible in Additional file 2: Table S2.

### Statistical analyses

The raw genotyping data were imported into GenomeStudio V2011.1 (Illumina) for SNP clustering and the generation of genotype calls. The standard summary statistics used for quality control of the genotyping were performed using PLINK [37].

We excluded 22 samples with an overall call rate < 90% and 34 SNPs with a call rate < 90%. The deviation of the genotype proportions from Hardy-Weinberg equilibrium (HWE) was assessed in the controls using Chi-squared test with one degree of freedom. Doing so, 3 SNPs with *p*-values < 0.001 showing significant deviations from HWE and were removed. This resulted in the inclusion of a total of 141 SNPs and 329 subjects (75 cases and 254 controls) in the analyses.

Conditional logistic regressions accounting for radiation dose to the thyroid were performed with PLINK [37] to assess the contribution of genetic factors to PTC risk. SNPs were included as a log additive model (i.e. multiplicative model of inheritance), which assumes the same increment in risk for each allele at a given locus. Dominant and recessive models were also examined for SNPs showing significant association in the log-additive model. Multiple testing was adjusted for using a Bonferroni correction after linkage disequilibrium (LD)-pruning to omit highly correlated SNPs, i.e. SNP pairs with r^2^ ≥ 0.8. After LD pruning, the number of tested markers was reduced to 94 SNPs.

As previously described, radiation doses were transformed as log(1 + dose), with the raw dose measured in Gy, in order to approximate the linear excess risk model for small doses in the conditional logistic regression [26]. In that way, “for the rare disease approximation, the relative risk of disease is modelled as approximately (1+dose)^β^, which for doses less than 1 Gy is approximately 1 + β dose” [26].

In addition to the single-marker-based association tests with PLINK, we employed the Versatile Gene-based Association Study (VEGAS) [38] and PLINK set [37] methods to examine whether test statistics for a group of related SNPs or genes have consistent yet moderate deviation from chance. Both VEGAS and PLINK set-based test combine *p*-values from single-SNP analyses but differ in how an appropriate null distribution is obtained. The gene-based association test performed by VEGAS relies on simulation of LD structure from a reference data set (here we used our control set) whereas PLINK set-based tests resort to permutation testing. For VEGAS, we used as input data the SNP association *p*-values obtained from the PLINK SNP-based logistic test, and the studied controls (option “poptem”) to estimate LD structure within each gene. The set test in PLINK is related to that used for pathway analysis; however here we used it only for genes and DNA repair module-based analysis. It calculates the average of all test statistics as a module enrichment scores, using independent and significant (by preselecting *p*-value cut-off) SNPs in the module [39]. PLINK set test generates empirical *p*-values using the max (T) permutation approach for pointwise estimates. The significance level of each gene was obtained through 10,000 permutations.

## Results

### SNP-based analysis

Out of the 407 study participants (83 cases and 324 controls) with blood DNA available (Table 1), 75 PTC patients and 254 matched controls were successfully genotyped using the Illumina SNP Cancer Panel array. Among those, 12 cases and 16 controls had received ionizing radiation doses above 2 Gy, and were excluded from the analyses because data were too sparse to allow proper fitting of the model described previously [26]. Doing so, analysis was restricted to 63 (84%) cases and 238 (93.7%) controls.

A total of 141 SNPs located in 43 DNA repair genes were present on the SNP Cancer Panel Array, passed the genotyping quality controls, were in agreement with HWE in controls (*P* > 0.001) and had a MAF in the control group greater than 0.05. The 43 genes containing these SNPs are involved in distinct DNA mechanisms that can be organized into 10 functional modules, as described in the ACSN [36]. The distribution of these 43 genes, as well as the number of tested SNPs, per functional module is shown in Table 2. As indicated in the legend of Table 2 some of the tested genes are involved in several DNA repair modules.

**Table 2:** Distribution of the 43 DNA repair genes per DNA repair module as described in the ACSN. The number of tested SNPs per group of genes is indicated in italic and in brackets

	NER	BER	MMR	SSA	NHEJ	MMEJ	HR	FANCONI	DR	TLS
NER	14 *[30]*									
BER	8 *[19]*	17 *[47]*								
MMR	3 *[9]*	6 *[21]*	10 *[47]*							
SSA	1 *[2]*	1 *[9]*	2 *[13]*	4 *[16]*						
NHEJ	-	1 *[5]*	-	-	4 *[13]*					
MMEJ	5 *[8]*	3 *[4]*	2 *[2]*	1 *[2]*	-	7 *[13]*				
HR	-	2 *[10]*	1 *[1]*	1 *[1]*	1 *[5]*	2 *[5]*	13 *[47]*			
FANCONI	2 *[4]*	-	-	1 *[2]*	-	2 *[4]*	4 *[21]*	7 *[34]*		
DR	-	-	-	-	-	-	-	-	1 *[4]*	
TLS	1 *[3]*	1 *[3]*	1 *[3]*	-	-	-	2 *[9]*	3 *[18]*	-	4 *[21]*

*NER* nucleotide excision repair, *BER* base excision repair, *MMR* mismatch repair, *SSA* Single strand annealing, *NHEJ* non-homologous end joining, *MMEJ* microhomology-mediated end joining, *HR* homologous recombination, *FANCONI* Fanconi pathway, *DR* Direct repair, *TLS* translesion synthesis

The 43 tested genes per DNA repair modules, with number of tested SNPs per gene indicated in brackets, are the following (some genes act in several modules): NER: *ERCC1* [2], *ERCC2* [2], *ERCC3* [2], *ERCC4* [2], *ERCC5* [2], *ERCC6* [2], *LIG1* [5], *LIG3* [1], *PCNA* [3], *POLD1* [1], *RAD23B* [3], *XPA* [1], *XPC* [2], *XRCC1* [2]; BER: *APEX1* [1], *ERCC5* [2], *LIG1* [5], *LIG3* [1], *MBD4* [1], *MLH1* [2], *MSH2* [9], *MSH6* [1], *OGG1* [2], *PARP1* [5], *PCNA* [3], *POLB* [2], *POLD1* [1], *RAD23B* [3], *WRN* [5], *XPC* [2], *XRCC1* [2]; MMR: *EXO1* [1], *LIG1* [5], *MLH1* [2], *MSH2* [9], *MSH3* [4], *MSH6* [1], *PCNA* [3], *PMS1* [18], *PMS2* [3], *POLD1* [1]; SSA: *ERCC1* [2], *MSH2* [9], *MSH3* [4], *RAD52* [1]; NHEJ: *LIG4* [1], *PARP1* [5], *XRCC4* [3], *XRCC5* [4]; MMEJ: *ERCC1* [2], *ERCC4* [2], *EXO1* [1], *LIG3* [1], *NBN* [4], *POLD1* [1], *XRCC1* [2]; HR: *BARD1* [1], *BLM* [4], *BRCA1* [7], *BRCA2* [5], *BRIP1* [5], *EXO1* [1], *NBN* [4], *PARP1* [5], *RAD51* [7], *RAD52* [1], *RAD54L* [1], *WRN* [5], *XRCC3* [1]; FANCONI: *BLM* [4], *BRCA1* [7], *BRCA2* [5], *BRIP1* [5], *ERCC1* [2], *ERCC4* [2], *FANCA* [9]; DR: *MGMT* [4]; TLS: *BLM* [4], *BRIP1* [5], *FANCA* [9], *PCNA* [3]

Results of the single-marker association test for the 7 SNPs showing the strongest association in the log-additive model (*P*_per allele_ < 0.05) are presented in Table 3. These SNPs are located in *MGMT, XRCC5, ERCC5, PARP1, PCNA, PMS2* and *OGG1* acting in 8 distinct DNA repair mechanisms. However, after adjustment for multiple testing, only the intronic SNP rs2296675 in *MGMT* acting in the DR module was significantly associated with PTC risk (Table 3). Other genetic models were also further examined for these 7 SNPs but associations did not reach statistical significance in these subsequent analyses. We also conducted a sensitivity analysis including the 28 subjects who received radiation doses above 2 Gy and results were similar (data not shown). Results of the association tests using different genetic models for the 141 DNA repair SNP and without including radiation dose in the models, are presented in Additional file 2: Table S2.

**Table 3:** Single marker associations with PTC (only SNPs with *P*_per allele_ < 0.05 are shown)

Gene	DNA repair module	SNP	Nucleotide change	Location relative to gene	MAF in cases	MAF in controls	Genetic model	OR (95% CI) ^a^	*P* ^*a*^	*P*_*corr*_^*b*^
*MGMT*	DR	rs2296675	A > G	Intronic	0.21	0.10				
							Risk per G allele ^c^	2.54 (1.50, 4.30)	0.0006	0.05
							A/G + GG versus A/A ^d^	2.72 (1.48, 5.03)	0.001	0.13
							G/G versus A/G + AA ^e^	5.30 (1.25, 22.48)	0.02	1
*XRCC5*	NHEJ	rs1051685	A > G	3’UTR	0.09	0.16				
							Risk per G allele ^c^	0.39 (0.20, 0.78)	0.008	0.73
							A/G + GG versus A/A ^d^	0.39 (0.18, 0.83)	0.01	1
							G/G versus A/G + AA ^e^	N/A	N/A	N/A
*ERCC5*	BER, NER	rs1047768	C > T	Coding (p.His46His)	0.49	0.39				
							Risk per T allele ^c^	1.58 (1.06, 2.35)	0.02	1
							C/T + T/T versus C/C ^d^	1.89 (1.05, 3.42)	0.03	1
							T/T versus C/T + CC ^e^	1.80 (0.88, 3.66)	0.11	1
*PARP1*	BER, HR, NHEJ	rs747659	C > T	Intergenic	0.12	0.18				
							Risk per T allele ^c^	0.49 (0.26, 0.94)	0.03	1
							C/T + T/T versus C/C ^d^	0.49 (0.25, 0.97)	0.04	1
							T/T versus C/T + CC ^e^	N/A	N/A	N/A
*PCNA*	BER, MMR, NER, TLS	rs17349	C > T	5’UTR	0.14	0.08				
							Risk per T allele ^c^	1.98 (1.07, 3.68)	0.03	1
							C/T + T/T versus C/C ^d^	1.93 (0.96, 3.85)	0.06	1
							T/T versus C/T + CC ^e^	8.29 (0.88, 78.3)	0.06	1
*PMS2*	MMR	rs3735295	G > A	Intronic	0.12	0.19				
							Risk per A allele ^c^	0.52 (0.28, 0.97)	0.04	1
							G/A + A/A versus G/G ^d^	0.49 (0.24, 1.00)	0.05	1
							A/A versus G/A + A/A ^e^	0.33 (0.05, 2.07)	0.24	1
*OGG1*	BER	rs125701	G > A	Intergenic	0.27	0.21				
							Risk per A allele ^c^	1.65 (1.01, 2.68)	0.04	1
							G/A + A/A versus G/G ^d^	1.87 (1.03, 3.40)	0.04	1
							A/A versus G/A + A/A ^e^	1.73 (0.47, 6.37)	0.41	1

When 2 or more SNPs in the same gene showed significant association, only the best SNP is reported

^a^ Dose was accounted for by including the continuous variable log(1 + dose). Subjects who received more than 2.0 Gy were excluded

^b^ *P*_*corr*_: Bonferroni corrected *p*-value

^c^ A log-additive model (i.e. a multiplicative model of inheritance), which assumes the same increment in risk for each allele at a given locus was used

^d^ Dominant model of inheritance (combined heterozygotes and rare homozygotes versus common homozygotes)

^e^ Recessive model of inheritance (rare homozygotes versus combined heterozygotes and common homozygotes)

*SNP* single nucleotide polymorphism, *MAF* minor allele frequency, *OR* odds ratio, *95% CI* 95% confidence interval, *UTR* untranslated region, *N/A* not applicable

### Gene-based analysis

We then conducted a gene-based analysis using VEGAS [38] which assigns SNPs to genes and calculates gene-based empirical association *p*-values while accounting for the LD structure within a gene. Using this approach, two genes, namely *ERCC5* and *MGMT* were significantly associated (*P*_Gene_ < 0.05) with PTC susceptibility (Table 4), suggesting that DR, BER, and NER DNA repair mechanisms may all play a role in the development of thyroid cancer. We also used PLINK set-based test to test each of the 10 DNA repair modules and observed significant association after Bonferroni correction for module DR (data not shown).

**Table 4:** Results of the gene-based association test from VEGAS (only genes with *P*_Gene_ < 0.05 or best SNP *P*_per allele_ < 0.05 are shown)

Chr	Gene	Start	Stop	Number of tested SNPs	Number of simulations	Test	*P*_Gene_	Best SNP	*P*_per allele_
10	*MGMT*	131,215,453	131,615,783	4	1e + 05	13.93	0.008	rs2296675	0.0006
13	*ERCC5*	103,448,190	103,578,351	2	1e + 06	9.39	0.01	rs1047768	0.02
20	*PCNA*	5,045,598	5,157,268	3	1e + 05	9.30	0.05	rs17349	0.03
7	*PMS2*	5,962,869	6,098,737	4	1000	8.70	0.09	rs3735295	0.04
1	*PARP1*	226,498,391	226,645,801	5	1000	11.05	0.11	rs747659	0.03
3	*OGG1*	9,741,627	9,858,353	2	1000	4.30	0.13	rs125701	0.05
2	*XRCC5*	216,924,019	217,121,016	4	1000	7.79	0.13	rs1051685	0.008

*Chr* chromosome, *Start* coordinate of the 5′ end of the gene on the chromosome, *Stop* coordinate of the 3′ end of the gene on the chromosome.

## Discussion

Understanding the aetiology of PTC and increased susceptibility to exposure to ionising radiation is an important aim for radiation protection policy. Radiation exposure during childhood is a strong risk factor for PTC, and polymorphisms in DNA repair genes are likely to affect this risk, but few studies have been designed to determine the role of such genes as modulators of PTC risk [40]. For this reason we chose to evaluate the association between a panel of common candidate SNPs located within 43 DNA repair genes and PTC. Our results showed significant association for rs2296675 located in *MGMT* encoding the O^6^-methylguanine DNA methyltransferase, and suggestive association for variants in *ERCC5* encoding a single-strand specific DNA endonuclease and variants in *PCNA* encoding the proliferating cell nuclear antigen. Hence our findings support the involvement of several mechanisms that could be mobilised by the follicular cells of the thyroid gland to repair the different types of DNA damages that could occur after exposure to radiation.

There are several limitations to this study that should be noted. First, the power to detect genes with small effect sizes may be low due to the relatively small number of subjects included in this study because of the uniqueness of the studied population. Indeed for SNPs with low MAF in controls (5%), the power of our study for evidencing an association between a candidate SNP and PTC could reach 80% only for an OR of 3.5 or higher for *P* < 0.05. For SNPs with MAF in controls of about 20%, our study had a power of 80% for evidencing an association if OR is about 1.90 *P* < 0.05.

Second, the genotyped markers in the Illumina SNP Cancer Panel array are scarce (on average 3.6 SNPs per cancer gene on the array, and 4.1 SNPs per DNA repair) and do not cover all of variations in the candidate genes. In addition, due to small sample size, we only included markers with a MAF ≥ 0.05, and common SNPs usually have small effects. Since the most significant SNPs that we identified are non-conding variants further sequencing and functional studies are required to confirm whether the disease associations of reported markers are causal.

Nevertheless, the association found between *MGMT* and PTC susceptibility is a novel interesting finding. MGMT is known to be one of the most important DNA repair proteins, and it catalyzes the transfer of the methyl group from O^6^-methylguanine adducts of double-stranded DNA induced by the alkylating agents to the cysteine residue in its own molecule and thus prevents the transition from G:C to A:T point mutations by removing alkyl adducts from the O^6^ position of guanine*.* Loss of MGMT expression has been associated with aggressive tumour behaviour and progression in several types of neoplasia, including esophageal, hepatocellular, lung, gastric and breast carcinomas [41–44]. The gene that is located at chromosome 10q26 spans nearly 300 kb of genomic DNA, where heterozygous deletion can often be observed in glioblastoma multiform patients [45].

Interestingly, the minor allele G of rs2296675 in *MGMT* had been previously shown to increase overall cancer risk of cancer across multiple tissues [per minor allele OR = 1.30, 95% CI 1.19,1.43, *P* = 4.1 × 10^−8^] [46], but risk of thyroid cancer was not specifically examined in the published CLUE II cohort study. Nevertheless, a link between MGMT and thyroid cancer had already been established in few studies. First, it was shown that expression level of MGMT protein was significantly downregulated in malignant compared to benign thyroid lesions [47]. Secondly, the methylation status of MGMT has also been investigated in thyroid neoplasia [48, 49]. Correlation of MGMT expression and promoter methylation with genomic instability in PTC patients had been then reported [50]. Functional studies are needed to decipher the biological properties of the methyltransferase and to understand how sequence variants may alter its functions and lead to the development of PTC. To our knowledge, the suggestive association between *PCNA* and PTC risk has not been observed in other populations, while a protective effect for carriers of the minor allele of rs2227869 in *ERCC5*, a SNP that is not present on the SNP Cancer Panel array, was reported in the Portuguese population [51]. Hence replication of the association study with a focus on *MGMT*, *ERCC5* and *PCNA* SNPs in other populations, including both irradiated and non-irradiated PTC patients and matched healthy subjects is also warranted. More widely, identification of genetic modifiers of radiation-associated carcinogenesis may thus be a step forward to allow future personalized cancer risk prediction and may serve in prevention endeavours or public health response to widespread radiation exposure.

## Conclusions

To conclude with, this study confirms that genetic variants in several genes operating in distinct DNA repair mechanisms are implicated in the development of PTC. In particular we report a new association between the minor allele G of SNP rs2296675 in *MGMT* and PTC risk in a unique population sample of Belarusian subjects who have been exposed to ionizing radiation during childhood. Further investigation is needed to decipher the functional properties of the methyltransferase encoded by this gene in order to understand how alteration of such functions may lead to the development of the most common type of thyroid cancer.

## Additional files

Additional file 1: Table S1. Annotation of the 1421 SNPs present on the Illumina SNP Cancer Panel array. (XLS 302 kb)

Additional file 2: Table S2. Results of the single marker association tests for each of the 141 DNA repair-related SNPs with MAF ≥ 0.05 present on the SNP Cancer Panel array which have passed genotyping quality controls. (XLS 145 kb)

## Abbreviations

BER: Base excision repair
DDR: DNA damage response
DR: Direct repair
DSBR: Double strand break repair
DTC: Differentiated thyroid carcinoma
FTC: Follicular thyroid carcinoma
GWAS: Genome-Wide Association Study
HWE: Hardy-Weinberg equilibrium
MAF: Minor allele frequency
MMR: Mismatch repair
NER: Nucleotide excision repair
PTC: Papillary thyroid carcinoma
QCs: Quality controls
SNP: Single nucleotide polymorphisms
TC: Thyroid cancer
